# Components of Adult Class III Malocclusion in an Iranian Population

**DOI:** 10.5681/joddd.2009.006

**Published:** 2009-03-16

**Authors:** Roodabeh Koodaryan, Ali Rafighi, Ali Hafezeqoran

**Affiliations:** ^1^Post-graduate student, Department of Prosthodontics, Faculty of Dentistry, Shahid Beheshti University of Medical Sciences, Tehran, Iran; ^2^Assistant Professor, Department of Orthodontics, Faculty of Dentistry, Tabriz University of Medical Sciences, Iran; ^3^Assistant Professor, Department of Prosthodontics, Faculty of Dentistry, Tabriz University of Medical Sciences, Iran

**Keywords:** Class III malocclusion, craniofacial abnormalities, protrusion, retrusion

## Abstract

**Background and aims:**

Class III malocclusions are considered complex and difficult orthodontic problems to diagnose and treat. The purpose of this study was to investigate the morphologic characteristics of the craniofacial complex of adults with Class III malocclusion in an Iranian population.

**Materials and methods:**

Lateral cephalometric radiographs of 50 patients with Class III malocclusion (20 males and 30 females; age range of 18-27 years) were selected on the basis of molar relationship, concave profile and an overjet of ≤ 0 mm. The standard values of 19 soft tissue measurements were determined using McNamara, Burstone and Viazis methods.

**Results:**

Adults with Class III malocclusion exhibited distinct craniofacial morphologic characteristics manifested by a com-bination of retrusion of maxilla and protrusion of mandible.

**Conclusion:**

The most prevalent component was mandibular prognathism, normal maxilla and LAFH on the basis of the component analysis.

## Introduction

Class III malocclusions are complex and difficult orthodontic problems to diagnose and treat. The prevalence of this type of malocclusion in white popu-lation is less than 5% but rises to as much as 12% in Chinese and Japanese populations. ^1 - 9^ Numerous stud-ies have been conducted to determine the morphologic variability of craniofacial complex in patients with Class III malocclusion. These studies have shown that the term Class III malocclusion is not a single diag-nostic entity but can consist of numerous combina-tions of skeletal and dentoalveolar components. ^10 - 15^

Sanborn found that 45.2% of his sample of adults with Class III malocclusion had mandibular skeletal protru-sion with orthognathic maxilla, and approximately 33% of the subjects had maxillary skeletal retrusion with the mandible within the normal range of protru-sion. A combination of maxillary skeletal retrusion and mandibular skeletal protrusion was observed in approximately 9.5% of the subjects. ^10^

Dietrich ^11^ showed in a study that the incidence of pure mandibular prognathism increased from 23% in the primary dentition to 31% in the permanent denti-tion. The corresponding figures for pure maxillary skeletal retrusion in deciduous, mixed and permanent dentition were 26%, 44% and 37%, respectively.

Ellis & McNamara^12^ analyzed a sample of Class III adult individuals who were selected on the basis of molar relationship. Pure maxillary retrusion and pure mandibular prognathism were found in 19.5% and 19.2% of the subjects, respectively.

Guyer et al^16^ evaluated a sample of patients with Class III malocclusion, who were aged 5 to 15 years. The authors divided the subjects into 4 consecutive age groups and compared them with Class III malocclusion with serial Class I control subjects. Analysis of the skeletal component combinations showed that 25% of the class III subjects had pure maxillary skeletal retrusion whereas less than 20% had pure mandibular prognathism.

Mouakeh^17^ investigated the craniofacial structure in a sample of 5 to 12-year-old Syrian children. Their results showed that 22% of the Class III samples (the most common) had maxillary retrusion with the mandible in normal range of protrusion and decreased lower anterior facial height (LAFH).

From the above-mentioned argument it can be concluded that Class III malocclusion can exist with a number of combinations of skeletal and dentoalveolar components within the facial skeleton. The aim of this study was to identify the morphologic characteristics of craniofacial structures in an adult Iranian population with Class III malocclusion.

## Materials and Methods

The subjects consisted of 50 patients (30 females and 20 males) with Class III malocclusion, with an age range of 18–27 years and a mean age of 22.56 ± 2.65 years. The patients were randomly selected from the retreatment orthodontics files of the Department of Orthodontics, Faculty of Dentistry, Tabriz University of Medical Sciences and private clinics in Tabriz. Inclusion criteria were:

- good quality pretreatment lateral cephalograms made on the same cephalostat (Planmeca Promax, Finland) with a magnification factor of 0.9
- Class III relationship of the first molars, determined by clinical evaluation of each patient in centric relation to rule out functional Class III malocclusion
- concave profile;
- an overjet of ≤ 0 mm
- no cleft palate or other craniofacial anomaly

A Class I control group matched for age and sex was generated from normative data reported for a similar population in a previous study.^18^ Thus, for each set of cephalometric measurements taken from the subjects with Class III malocclusion, an equivalent set was derived from the normative data tables with controlled age and sex. Therefore, there was no ethical consideration in this regard.

Lateral cephalograms were traced, and cephalometric reference points were determined with the use of a 0.5-mm lead pencil on acetate tracing paper.

Twenty-seven linear and angular measurements were made. The standard values of 19 soft tissue measurements were determined using McNamara, Burstone and Viazis methods. The means and standard deviations for the Class III subjects were calculated with the use of the SPSS computer program (SPSS Inc, Chicago, Il3). Independent sample t-test (if data were normally distributed) and U Mann-Whitney (when data were not normally distributed) were used to assess the differences between the Class III group and the controls. A P-value of 0.05 or more was considered not significant.

## Results

To determine the frequency with which the various skeletal components occurred in the Class III subjects, a neutral range for individual measures of maxillary and mandibular skeletal positions and vertical facial dimension was selected (Table 1).

**Table 1:** Comparison of linear and angular measurement of Class III adults and norms using independent sample t-test and U Mann-Whitney statistical test

		Class I N = 50	Class III N = 50		Asymptotic significance	
Component	Variables	Mean	SD	Mean	SD	Z Value	(2-tailed)	Significance
Cranial base	S-N (mm)	76.63	4.062	72.84	4.542	-3.398	0.000	***
	S-Ar (dg)	37.92	37.920	34.24	3.378	-5.380	0.000	***
	N-S-Ar (dg)	124.84	3.672	121.56	3.855	-3.829	0.000	***
	N-S-Ba (dg)	129.58	4.500	128.40	5.127	-0.788	0.431	NS
	SN-FH (dg)	6.71	2.263	8.20	2.949	-2.612	0.009	**
Maxillary	A-N perp (mm)	-1.66	2.110	-3.65	3.540	-3.398	0.001	**
	SNA (dg)	81.20	2.626	79.20	2.733	0.001	0.001	**
	Co-A (mm)	94.46	5.779	87.74	5.952	-5.025	0.000	***
Mandibular	Pog-N perp (mm)	-4.10	1.773	1.12	6.150	-4.820	0.000	***
	B-N perp (mm)	-4.94	1.406	0.37	5.948	-5.253	0.000	***
	SNB (dg)	78.56	1.831	82.17	3.350	-5.731	0.000	***
	GoMe/FH (dg)	23.94	3.383	27.84	5.936	-4.243	0.000	***
	GoGn/SN (dg)	31.90	4.229	35.84	5.626	-4.216	0.000	***
	Co-Gn (mm)	123.24	8.098	130.14	7.804	-3.982	0.000	***
	Facial angle (dg)	85.60	2.399	90.02	3.583	-6.002	0.000	***
Intermaxillary	ANB (dg)	2.96	1.564	-2.85	2.726	-7.927	0.000	***
	PP/Go-Me (dg)	24.00	3.010	28.32	5.923	-4.778	0.000	***
Dentoalveolar	U1-A perp (mm)	4.91	1.484	6.21	2.900	-2.485	0.013	*
	U1/PP (dg)	109.36	3.674	114.92	7.442	-4.700	0.000	***
	U1/SN (dg)	101.08	3.325	105.90	8.026	-3.770	0.000	***
	L1/A Pog (mm)	3.32	1.728	6.41	2.298	-6.497	0.000	***
	L1/A Pog (dg)	25.82	3.486	23.30	6.011	-2.480	0.013	*
	IMPA (dg)	96.48	4.348	79.26	8.022	-8.186	0.000	***
	Overjet (mm)	2.29	0.701	-2.21	2.241	-8.632	0.000	***
	U1/L1 (dg)	130.86	8.144	139.72	10.622	-4.275	0.000	***
Facial height	ANS-Me (mm)	72.70	5.905	75.74	6.375	-2.317	0.021	*
	LAFH/TAFH	0.55	0.018	0.56	0.024	-0.885	0.376	NS

NS = Not Significant (P > 0.05); *: P < 0.05; **: P < 0.01; ***: P < 0.001

The neutral range for measures was established from the control group as the mean value ± 1 standard deviation. Values less than the neutral range indicated a retrusive position for the maxilla or mandible and decreased lower anterior face height. Values greater than the neutral range indicated a protrusive position of maxilla or mandible or increased lower anterior facial height. Each value could then be classified as low, neutral, or high.

To clarify the most common setting for Class III patients, two series of classifications were used. The first classification was done on the SNA angle, facial angle, and lower anterior facial height (Table 2). With this classification, the patients were divided into 10 groups. The most common combination (28%) was prognatic mandible with normal maxilla and facial height. The patients were divided into 11 group in the second classification based on the liner distance of A and Pog from Nasion perpendicular and lower facial height (Table 3). The most common set (22%) in this classification was protrusive mandible, and normal maxilla and lower facial height.

**Table 2:** Classification of patients on the basis of SNA angle, facial angle, and lower anterior facial height

Group	N	SNA	Facial angle (N Pog-FH)	LAFH	Percentage
1	14	=	→	=	28
2	11	=	→	↑	22
3	8	←	=	↑	16
4	6	=	=	=	12
5	3	←	→	↑	6
6	2	=	→	↓	4
7	2	←	=	=	4
8	2	=	=	↑	4
9	1	←	=	↓	2
10	1	←	→	↓	2

←: retrusive; →: protrusive; ↓: decreased LAFH; ↑: increased LAFH; =: normal.

**Table 3:** Classification of patients on the basis of linear distance of A and Pog from Nasion perpendicular and lower anterior facial height

Group	N	A-N Prep	Pog-N Prep	LAFH	Percentage
1	11	=	→	=	22
2	8	←	=	↑	16
3	7	←	→	↑	14
4	7	=	→	↑	14
5	5	←	→	=	10
6	5	←	=	=	10
7	2	=	→	↓	4
8	2	=	=	↑	4
9	1	←	=	↓	2
10	1	←	→	↓	2
11	1	=	=	=	2

←: retrusive; →: protrusive; ↓: decreased LAFH; ↑: increased LAFH; =: normal.

## Discussion

Class III malocclusion has long been viewed as one of the most severe facial anomalies. It is now well established that this malocclusion is not limited to dental discrepancies but is often related to an underlying skeletal problem.^19^ A developing Class III malocclusion can exhibit maxillary skeletal retrusion, mandibular skeletal protrusion, or some combination of the two. In addition, an excessive or deficient vertical facial dimension is frequently displayed and will have an impact on the diagnostic and therapeutic decisions.

This study was undertaken to determine the morphologic characteristics of the craniofacial complex of adults with Class III malocclusion. The most interesting finding was the high prevalence of mandibular skeletal protrusion among the patients with Class III malocclusion. The mandibular position was within neutral range of protrusion, although some indicators of mandibular overdevelopment were observed in patients with Class III malocclusion.

Dental aberrations were represented by a significant protrusion and labial inclination of maxillary incisors whereas mandibular incisors showed lingual inclination.

Analysis of the various components of Class III malocclusion of the adults included in this study confirmed that most of them exhibited a normal maxilla and prognathic mandible. In the present study this was seen in 54% of cases, which is in accordance with the results of Sanborn^10^ and Jacobson^20^ for adults but more than the results of studies by Ellis et al^12^ and Dietrich^11^ in mixed dentition.

Maxillary pure retrusion was seen in 22% of the cases in the first classification and 28% of the subjects in the second classification. These findings are consistent with those reported by Guyer et al,^16^ Jacobson^11^ and Ellis et al.^12^

Combination of retruded maxilla and protruded mandible was seen in 8% of the cases with the first classification and 26% of the subjects with the second classification similar to the results reported by Sanborn^10^ and Jacobson.^20^ Considering facial height, most of the subjects with Class III malocclusion showed increased facial height and just 8% of cases had decreased facial height. These findings coincide with those of Guyer et al.^16^

Interpreting the results of both classifications showed that the most commonly found skeletal component in adults with Class III malocclusion is protrusive mandible with normal maxilla and facial height.
